# Genome-based classification of *Burkholderia cepacia* complex provides new insight into its taxonomic status

**DOI:** 10.1186/s13062-020-0258-5

**Published:** 2020-03-04

**Authors:** Yuan Jin, Jianglin Zhou, Jing Zhou, Mingda Hu, Qi Zhang, Na Kong, Hongguang Ren, Long Liang, Junjie Yue

**Affiliations:** 1 Beijing Institute of Biotechnology, No. 20, DongDaJie Street, Fengtai, Beijing, 100071 China; 2 State Key Laboratory of Pathogen and Biosecurity, No. 20, DongDaJie Street, Fengtai, Beijing, 100071 China; 3 Anhui University, Hefei, 230039 Anhui China

**Keywords:** *Burkholderia cepacia* complex, Genome, Taxonomy, Classification, Single-copy orthologous genes, dDDH and ANI

## Abstract

**Background:**

Accurate classification of different *Burkholderia cepacia* complex (BCC) species is essential for therapy, prognosis assessment and research. The taxonomic status of BCC remains problematic and an improved knowledge about the classification of BCC is in particular needed.

**Methods:**

We compared phylogenetic trees of BCC based on 16S rRNA, *recA*, *hisA* and MLSA (multilocus sequence analysis). Using the available whole genome sequences of BCC, we inferred a species tree based on estimated single-copy orthologous genes and demarcated species of BCC using dDDH/ANI clustering.

**Results:**

We showed that 16S rRNA, *recA*, *hisA* and MLSA have limited resolutions in the taxonomic study of closely related bacteria such as BCC. Our estimated species tree and dDDH/ANI clustering clearly separated 116 BCC strains into 36 clusters. With the appropriate reclassification of misidentified strains, these clusters corresponded to 22 known species as well as 14 putative novel species.

**Conclusions:**

This is the first large-scale and systematic study of the taxonomic status of the BCC and could contribute to further insights into BCC taxonomy. Our study suggested that conjunctive use of core phylogeny based on single-copy orthologous genes, as well as pangenome-based dDDH/ANI clustering would provide a preferable framework for demarcating closely related species.

**Reviewer:**

This article was reviewed by Dr. Xianwen Ren.

## Background

*Burkholderia cepacia* complex (BCC) is a group of gram-negative bacteria comprising more than 20 valid species names, including *B. cepacia*, *B. multivorans*, *B. cenocepacia*, *B. vietnamiensis*, *B. stabilis*, *B. ambifaria*, *B. dolosa*, *B. anthina*, *B. pyrrocinia* and *B. ubonensis*, etc. [1–3]. Before the 1990s, the *Burkholderia cepacia* complex was simply known as one species, *Burkholderia cepacia*. Even *B. cepacia* was considered to be *Pseudomonas cepacia* when it was first isolated in patients with cystic fibrosis (CF) in 1977 [4]. In the mid-1990s, researchers noted that *B. cepacia* was, in fact, composed of multiple distinct subgroups, and five genomovars were initially identified: *B. cepacia* (genomovar I), *B. multivorans* (genomovar II), *B. cenocepacia* (genomovar III), *B. stabilis* (genomovar IV), *B. vietnamiensis* (genomovar V) [5]. Thus, *B. cepacia* is not a single species but should be named the *B. cepacia* complex, which consists of multiple phenotypically similar but genetically distinct microorganisms. Subsequently, studies identified large heterogeneity among BCC bacteria, leading to more species, such as *B. ambifaria* and *B. pyrrocinia*, were added to this complex [6, 7]. The *B. cepacia* complex bacteria have been reported as opportunistic pathogens that caused pneumonia in people with cystic fibrosis (CF) or chronic granulomatous disease [8]. These organisms are associated with accelerated decline in pulmonary functions, increasing morbidity and mortality and reducing survival following lung transplantation [9]. Patients infected with BCC bacteria may develop syndromes associated with septicemia, which is associated with high mortality. BCC is noted for its different resistance mechanisms, which confer nonsusceptibility to most of the available antibiotics, making infections very difficult to eradicate [10]. Furthermore, outbreaks of different BCC species are often reported, and there is a large body of evidence showing that BCC bacteria are capable of patient-to-patient spread [11].

In light of the significance of the BCC, species identification and taxonomy of the isolates within the complex are of great importance. For example, infection with BCC can be considered a contraindication for lung transplantation due to increased mortality, but the increase in risk appears to vary significantly from species to species [12]. Therefore, differentiation of BCC species is helpful for clinical therapy, prognosis assessment and epidemiological research. However, correct identification of these pathogens can be particularly problematic because they have a high level of similarity. Phenotypic identification involving either manual or automated systems such as Phoenix, VITEK 2, and VITEK MS. cannot give reliable results, and studies have shown that phenotypic tests are not suitable for the identification of these pathogens [13, 14]. Developing molecular biology methods such as PCR and sequence analysis of targets such as 16S rRNA, *recA* and *hisA* are used to identify bacteria in this complex [15, 16]. However, few large-scale studies have attempted to comprehensively evaluate the power of these markers. Multilocus sequence typing/analysis (MLST/MLSA), which utilizes nucleotide sequences of multiple alleles, including *atpD*, *gltB*, *gyrB*, *recA*, *lepA*, *phaC* and *trpB* genes, showed improved power in discriminating the species belonging to this complex [17]. Nevertheless, as there is great genetic diversity between BCC bacteria, many STs are still not well characterized [13].

Due to the great likeness between BCC bacteria and the lack of accurate identification methods with high sensitivity and specificity, the taxonomic status of the *B. cepacia* complex remains unknown. Phylogenetic analysis based on the *recA* allele shows that the medically important BCC member *B. cenocepacia* comprises four lineages (referred to as *B. cenocepacia* genomovars IIIA, IIIB, IIIC and IIID). *B. cenocepacia* IIIE was described in MLST/MLSA studies yet was suggested to be a misassignment later on [18]. Other BCC groups also complicate the classification. For example, the former taxon K, a group within the *Burkholderia cepacia* complex, contains at least two species now: *Burkholderia contaminans* and *Burkholderia lata* [19]. In addition to the well-established BCC species, an increasing number of clusters defined by previous research were proven to be novel species. For instance, the initial BCC group B was described as *B. stagnalis* and BCC group L as *B. territorii* [20]. Despite a great deal of efforts have done to reveal their taxonomic complexity, many BCC strains still have controversial species assignments, which is necessary. In addition, high phenotypic and genotypic similarity of different BCC groups often lead to misidentification, which might cause problems with patient therapy. All of these phenomena suggest that the identification and taxonomic relationships of bacteria within the BCC are controversial, and the classification of this complex is still not well elucidated. Therefore, the taxonomy and classification of the *B. cepacia* complex should be reassessed, and improved knowledge about the BCC is in particular needed.

At present, with the advent of next-generation sequencing technologies, an increasing number of complete genome sequences of the bacteria in the BCC have been published [21–24]. This provides an ideal opportunity for re-examining the taxonomy of BCC by traditional molecular methods. In addition, the whole genome sequencing (WGS) data enable us to identify pathogens and reveal the evolutionary pattern of the bacteria based on whole genome information rather than a single locus or combination of several loci. DNA:DNA hybridization (DDH) and whole genome average nucleotide identity (ANI) values have been widely applied as a gold standard for the prokaryotic species definition [25–27]. The coming digital DNA:DNA hybridization (dDDH) method, which uses whole genome sequences, can overcome many challenges of the tedious and complicated traditional DDH experiments [28]. In this study, a variety of traditional approaches, including 16S rRNA gene analysis, phylogeny based on the housekeeping gene *recA* and *hisA*, and MLSA were initially applied. In addition, with whole genome information, we determined more than 1000 single copy homology genes of BCC and estimated a more robust and resolved species tree of this complex. Then, we employed dDDH and ANI to systematically study the taxonomic status of the *B. cepacia* complex using whole-genome sequences. We compared the outcome of these approaches and explored the problematic taxonomic status and misidentification of bacteria within the BCC the analyses exposed. Furthermore, our study reconsidered the classification of BCC species mainly based on WGS-based approaches since these methods utilize a much larger part of the genome and have a better resolution for discriminating closely related bacteria [29]. The aim of the present study was (i) to contribute further insight into the taxonomy and phylogeny of BCC species, (ii) to suggest a reliable and relatively different view to demarcate bacteria in the BCC, and (iii) ultimately to obtain a more satisfactory classification of *Burkholderia cepacia* complex.

## Methods

### Whole-genome data set preparation

A data set of 255 BCC whole genomes with assembly levels of Complete Genome, Chromosome and Scaffold was obtained from the GenBank database on April 14, 2019 [30]. The quality estimates of these genomes were determined with CheckM using the lineage-specific workflow and default parameters [31]. A genome was included only if it had ≥ 90% completeness, ≤ 10% contamination and an overall quality ≥ 50% (defined as completeness - 5 * contamination) [32]. After filtering, the genomes were dereplicated as described in Parks et al. [33], except that the dereplication was based on the ANI values estimated by FastANI with default parameters [34]. After checking the quality and dereplication, a total of 112 Bcc genomes belonging to 22 Bcc species were kept for further analysis. Additionally, four BCC genomes with a Contig-level assembly were also included in our data set because they were assembled from type material and their species did not have a better genomes from the type strain. The four genomes passed a quality check as well. Detailed information and quality evaluation results of the 116 tested strains are presented in Additional file 1.

### Phylogenetic analysis of the 16S rRNA gene, *recA* gene and *hisA* gene

For the 116 tested strains, the full-length sequences of the 16S rRNA gene, *recA* gene and *hisA* gene were extracted from the genome sequences using BLASTN with the corresponding sequences of *Burkholder cenocepacia* J2315 as queries [17]. The full length 16S rRNA gene could not be extracted for six of the 116 strains; therefore, these six strains were removed from the phylogenetic analysis of the 16S rRNA gene, except one strain (*B. cepacia PT02*) with a 16S rRNA gene length longer than 1000 bp (Additional file 2). Additionally, eight 16S rRNA gene sequences from different type strains of BCC species, which lacked completely sequenced whole genomes, were downloaded from GenBank and included for analysis (Additional file 3). Pairwise distances were calculated via p-distances and using “Pairwise deletion” for gaps/missing data treatment. The *hisA* gene from only one of the 116 strains (*B. ubonensis* MSMB0106) could not be extracted and was removed from the phylogenetic analysis of the *hisA* genes. After extraction, all gene sequences were individually aligned using the Muscle program [35] and trimmed by trimAl with default parameters by which the positions with more 50% gaps were clipped [36]. A maximum-likelihood phylogenetic tree of each gene was generated by MEGA-X software, using the General Time Reversible model, G + I rates among sites and a bootstrap method with 1000 replications [37]. The trees and support values were visualized using iTOL [38].

### Phylogeny based on MLSA

A MLSA of the BCC strains was performed using seven housekeeping genes: *atpD*, *gltB*, *gyrB*, *recA*, *lepA*, *phaC* and *trpB* [19]. We downloaded seven housekeeping gene fragments of *Burkholder cenocepacia* J2315 from the PubMLST database (https://pubmlst.org/bcc/) as BLAST search queries [39]. For each gene fragment of each tested strain, we used the blastn program to extract the corresponding allelic fragments from the assembled genomes with an E-value cut-off of 1e-5. Extracted allelic fragments were aligned using the Muscle program [35] and trimmed by trimAl with default parameters by which the positions with more 50% gaps were clipped [36]. Seven multiple sequence alignments were then concatenated by AMAS [40] to infer maximum likelihood phylogeny with MEGA-X under the GTR model using G + I rates among sites and a bootstrap method with 1000 replications [37]. The phylogenetic tree and support values were visualized using iTOL [38].

### Reconstruction of species tree

Groups of orthologous sequences were defined using OrthoFinder2 [41] and aligned with MAFFT version 7.271 [42]. Each amino acid alignment was trimmed by trimAl [36] and then concatenated into a core-genome alignment by AMAS [40]. A maximum likelihood phylogeny of concatenated single-copy core-genome was inferred using FastTree version 2.1.11 with multithreading and the parameters “-gamma -spr 4 -wag -mlacc 2” [43]. The phylogenetic tree root was determined at the node that was pointed by the MLSA with outgroups. The phylogenetic tree and support values were visualized using iTOL [38].

### Average nucleotide identity (ANI) and digital DNA–DNA hybridization (dDDH) calculation

For the clarification of species affiliations, dDDH [26, 44] and ANI [25] were used for nucleotide-level comparisons for every pairwise combination of genomes. All pairwise ANI values of the tested strains were estimated using FastANI [34] with default parameters. The GC content of every strain was also estimated from their genomes during the ANI calculation. All pairwise dDDH values were calculated by GGDC 2.1 (Genome-to-GenomeDistance Calculator, http://ggdc.dsmz.de/distcalc2.php) under the recommended Formula 2 with the alignment tool BLAST+. Genome-to-Genomedistances (GGDs) of every two genomes of the 116 tested strains were calculated by GGDC 2.1 as well. Estimates for species affiliations were obtained by clustering the GGDs with the distance corresponding to 70% dDDH (0.0361 for the recommended GGDC setting) and nonhierarchical linkage clustering with an F value of 0.5 as implemented in OTPSIL [45]. As suggested by previous studies [28, 46], the F value of 0.5 yielded the highest clustering consistency for the present data at the predefined threshold. Similarly, we inferred genome distance (D) from ANI values by equation *D* = 1 − *ANI*, and then estimated the species affiliations by clustering D with the distance corresponding to 95 and 96% ANI (0.05 and 0.04 for D), respectively.

## Results

### Phylogenetic analysis based on single molecular markers

**Fig. 1:**
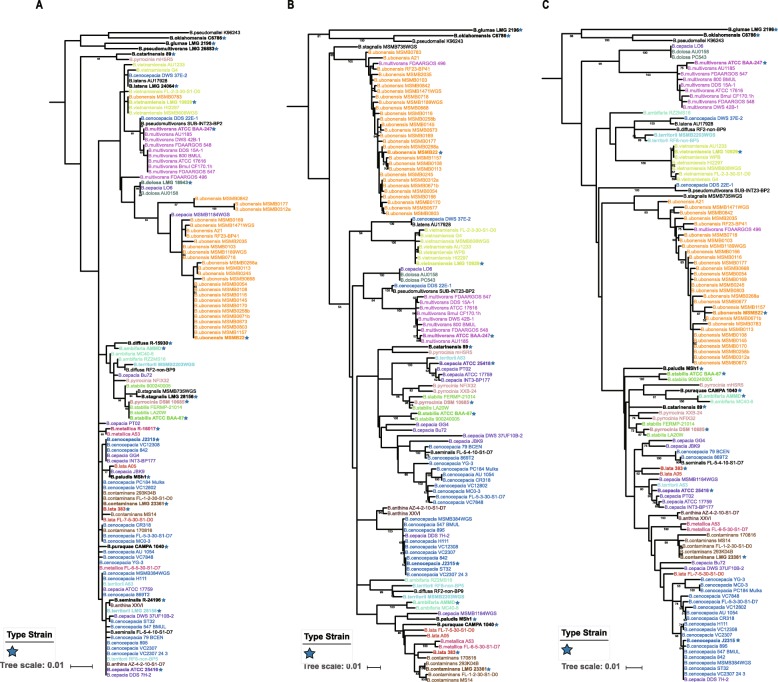
The phylogenetic relationships of BCC strains based on (A) 16S rRNA, (B) *recA* and (C) *hisA* sequences.The maximum-likelihood trees were constructed from the alignments of (**a**) full length 16S rRNA (1539 bp), (**b**) full length *recA* (1071 bp) and (**c**) full length *hisA* (756 bp) sequences. *B. pseudomallei* K96243, *B. oklahomensis* C6786^T^ and *B. glumae* LMG 2196^T^ were chosen as outgroups. Numbers below branches are bootstrap support values from 1000 replicates if equal to or larger than 50%. Type strains are printed in bold font as well as marked by a blue star (*B. ubonensis* MSMB22 is the representative genome of this species; because of its examined high-quality and unavailability of the whole genome of this species, we treated MSMB22 as the same as the type strain of *B. ubonensis* in this paper). Species with at least two members are colored except *B. pseudomultivorans*, *B. latens*, *B. diffusa*, *B. stagnalis* and *B. seminalis* in (**a**); species with single members (and outgroups) are black

The 16S rRNA, *recA* and *hisA* genes are widely used as molecular markers to study BCC bacteria. To determine the impact of using these genes for identifying the BCC taxa, we performed phylogenetic analyses using available sequences from all 116 BCC strains. We included three outgroup strains, *B. pseudomallei* K96243, *B. oklahomensis* C6786, and *B. glumae* LMG 2196, in the analysis and added as many related type strains as possible (Additional file 3). Five strains were removed from the 16S rRNA-based phylogenetic analysis due to failure to extract the full-length 16S rRNA gene sequences. The *hisA* gene could not be extracted from one of the 116 strains (*B. ubonensis* MSMB0106), and this strain was excluded from the *hisA* analysis. All *recA* sequences from the 116 BCC strains were included for the construction of *recA*-based phylogeny. The phylogenetic trees based on 16S rRNA, *recA* and *hisA* are shown in Fig. 1.

The phylogenetic tree of 16S rRNA shows a poor and low bootstrap support overall (Fig. 1a). Many clades were condensed and nested to each other, especially in those lineages comprising *B. cenocepacia*. These observations revealed that the amount of phylogenetic signal presented by 16S rRNA is small, resulting in many short internal branches that are difficult to resolve. Pairwise comparison of sequences from BCC stains revealed that their identical levels were between 97.87 and 100%. Similarities of the BCC strains towards three outgroups were also in the ranges of 97.91–99.09%. Notably, some type strains of different BCC species share a nearly identical 16S rRNA sequence, which leads to their relationship being completely unresolved. For example, the type strains *B. stabilis* ATCC BAA-67^T^ and *B. pyrrocinia* DSM 10685^T^ share identical 16S rRNA gene sequences. In addition, *B. lata* 383^T^ and *B. contaminans* LMG 23361^T^ also share identical 16S rRNA gene sequences with five other strains, including those taxonomically annotated by Genebank as *B. lata* FL-7-5-30-S1-D0, *B. contaminans* 293K04B, *B. contaminans* FL-1-2-30-S1-D0, *B. cenocepacia* PC184 Mulks and *B. cenocepacia* VC12802. These results indicate that the 16S rRNA gene has low taxonomic resolution in the identification of strains with BCC, which is in line with other reports [13, 14, 47].

In contrast, the phylogenetic trees inferred from the *recA* and *hisA* genes were well resolved (Fig. 1b and c). The lineages were divided and grouped clearly with branches showing very strong bootstrap support. Similarity analysis demonstrated that the average identical levels of both genes were slightly larger than 95%. Specifically, the *recA* sequences of 116 BCC strains showed a range of 93.08–100% identity, while the *hisA* sequences of 115 strains ranged from 91.76 to 100%. Although these two trees revealed a better resolution than the 16S rRNA-based phylogeny, the trees surprisingly exhibit some extent of confusion and discordance. In both phylogenies, strain *B. cepacia* DWS 37UF10B-2 is far from the *B. cepacia* major clade represented by the type strain *B. cepacia* ATCC 25416^T^ (Fig. 1b and c). In fact, *B. cepacia* DWS 37UF10B-2 is not clustered in any other taxa clade and forms a single branch. This phenomenon suggests that this strain probably represents a species different from the current BCC species in view of the *recA* and *hisA* phylogenies. In the *recA*-based phylogeny, strain *B. cepacia* GG4 formed a dependent branch different from the *B. cepacia* major clade with *B. cepacia* Bu72 (Fig. 1b). However, this strain formed a similar branch with *B. cepacia* JBK9 in the *hisA*-based phylogeny (Fig. 1c). The distinct independent branches suggest that the current taxonomic classification of *B. cepacia* may be problematic and need to be further divided. Similar situations can be observed in other BCC species, such as *B. cenocepacia* and *B. ambifaria*. Moreover, clade *B. cenocepacia* IIIA represented by *B. cenocepacia* J2315^T^ and *B. cenocepacia* IIIB represented by *B. cenocepacia* AU 1054 were separated into two different clades in the *recA*-based phylogeny but shared a recently common ancestor in the *hisA*-based phylogeny (Fig. 1b and c). The cluster and topology difference suggest that the phylogeny based on different individual genes may conflict due to their different evolutionary history. All these contradictions also demonstrated that single gene-based phylogeny could hardly reconstruct the true phylogenetic relationship of BCC species. Despite the discordance exhibited by the *recA* and *hisA* trees, many BCC strains seem to be misidentified by a previous study according to the concordant result from the two phylogenetic trees. For example, *B. multivorans* FDAARGOS 496 clustered in the *B. ubonensis* clade is more likely to be *B. ubonensis* rather than *B. multivorans*. Two isolates previously identified as *B. cenocepacia*, DDS 22E-1 and DWS 37E-2, are more likely to be *B. pseudomultivorans* and *B. latens*, respectively. *B. stabilis* LA20W seems to be more similar to the type strain *B. pyrrocinia* than *B. stabilis*, which was also confirmed by another study [1]. Two additional strains, LO6 and DDS 7H-2, were previously identified as *B. cepacia* and probably belong to *B. dolosa* and *B. cenocepacia*, respectively. *B. territorii* A63 may be *B. cepacia* because it is more similar to type the strain *B. cepacia* ATCC 25416 in both trees (Fig. 1b and c).

### Species tree based on genomes and comparison to MLSA

**Fig. 2:**
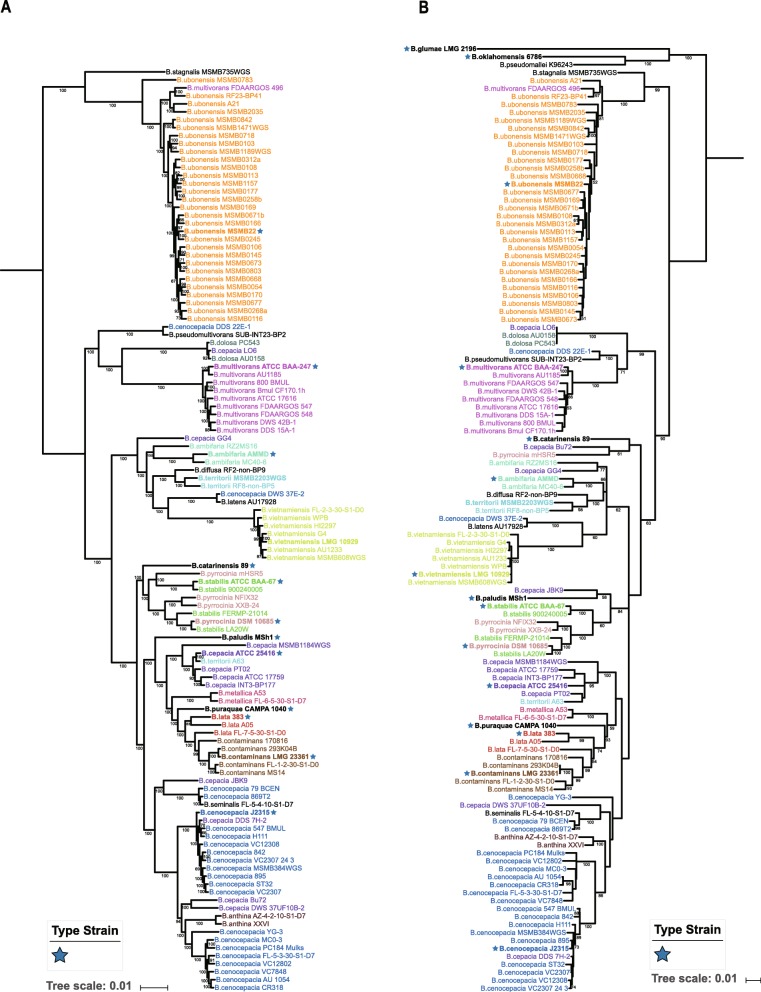
Comparison of MLSA and species tree. **a** Maximum-likelihood phylogenetic tree of 116 BCC genomes based on concatenated amino acid alignments of 1005 single-copy orthologous genes (274,980 AA) and rooted at node pointed out by MLSA phylogeny. Node support values were based on the Shimodaira-Hasegawa test. **b** Maximum-likelihood phylogenetic tree of the concatenated nucleotide sequences (2771 bp) from the seven housekeeping gene fragments [*atpD* (443 bp), *gltB* (400 bp), *gyrB* (454 bp), *recA* (393 bp), *lepA* (395 bp), *phaC* (385 bp) and *trpB* (301 bp)]. *B. pseudomallei* K96243, *B. oklahomensis* C6786^T^ and *B. glumae* LMG 2196^T^ were chosen as outgroups. Other display settings in (**a**) and (**b**) are the same as in Fig. 1

To overcome the defects of single molecular markers, we conducted a MLSA, which are widely used to differentiate BCC strains. Here, all seven loci (*atpD*, *gltB*, *gyrB*, *recA*, *lepA*, *phaC* and *trpB*) were successfully extracted for 114 of the 116 tested strains, and two strains at all but one (*B. ubonensis* MSMB0106 and MSMB0108). We still included these two strains for MSLA because the sequences from the other six genes were normally sufficient to identify a BCC isolate [11, 48]. The phylogenetic tree of seven concatenated housekeeping loci is shown in Fig. 2b.

To reconstruct the accurate genealogy of BCC species, we estimated a species tree using only single-copy orthologous genes. These genes are vertically inherited during evolution and thus preserved a more complete genealogical history. Trees inferred from the concatenation of single-copy protein sequences provide higher resolution than those obtained from a single phylogenetic-marker gene or multiple loci [32, 49, 50]. Studies have shown that using single-copy orthologous genes could minimize artifacts that result from the confounding effects of horizontal gene transfer [51, 52]. To recover the species tree of all BCC strains, a total of 1005 single-copy orthologous genes shared by all 116 strains were first identified. Then, we aligned each orthologous family and concatenated them to infer a maximum likelihood phylogeny (Fig. 2a).

The inferred species tree and the MLSA-based phylogenetic tree are displayed in Fig. 2. The MLSA tree and species tree were well resolved, and they revealed robust support with most branches having a maximum support value. The two trees showed a much more similar topology and consistent pattern with each other. Especially regarding the major clades and backbone branches, the MLSA phylogeny was completely congruent with the single-copy orthologous genes-based phylogeny. Although slight differences can be observed, strains of different BCC species were well organized and grouped regularly. Nine taxa, including *B. ubonensis*, *B. pseudomultivorans*, *B. dolosa*, *B. multivorans*, *B. latens*, *B. vietnamiensis*, *B. metallica*, *B. contaminans* and *B. anthina*, formed monophyletic groups with high support both in the MLSA phylogeny and species tree (Fig. 2). However, the cluster status of the two trees highlighted apparent taxonomic inconsistencies. First, the possible misidentification of the seven strains mentioned above was reconfirmed with the two phylogenies (Fig. 2). Second, the confusing circumstances were still observed between *B. cepacia*, *B. cenocepacia* and *B. ambifaria*, as well as between the *B. pyrrocinia* and *B. stabilis* groups.

### BCC species demarcation based on dDDH and ANI

**Fig. 3:**
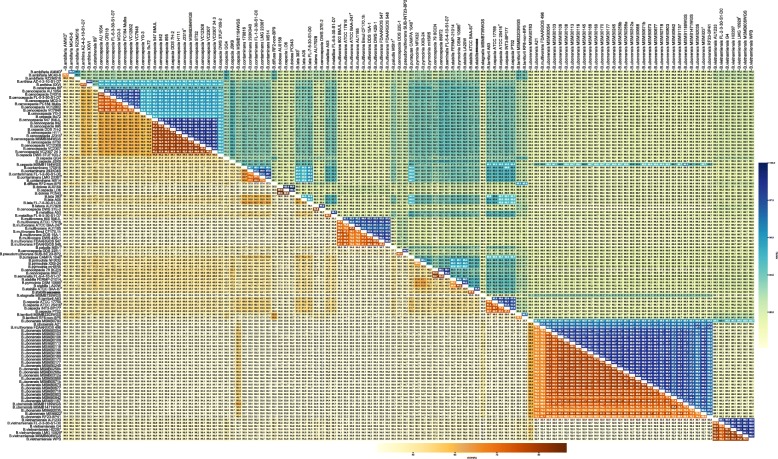
Heat map of dDDH and ANI from pairwise genomes comparisons.The lower triangle displays the dDDH values, and the upper triangle displays the ANI values. Type strains are indicated by a superscript T

Our phylogenetic analyses revealed the confusing status of current BCC taxonomy and the possible misidentifications. Though the relationship of the different BCC clades was provided, they could not determine the species boundaries. As complementary methods, DDH and ANI values are widely used as a gold standard for the prokaryotic species definition. These two approaches evaluate the whole genomic similarity of bacteria, and dDDH is a fast and accurate replacement for the traditional laboratory-based DDH [25, 26, 53]. Here, we performed in silico dDDH and ANI analyses based on whole genome sequences and the results are listed in Additional file 4. We primarily used the pairwise dDDH values to cluster BCC species and their corresponding ANI values to cross reference and evaluate the congruence of the two approaches (Fig. 3). Although dDDH and ANI use different algorithms for the calculations, i.e., ANI evaluates the similarity of two genomes from the shared elements or fragments, while dDDH uses the sequence similarity of conserved regions between two genomes [54], the results were very consistent (Additional file 5). The ANI values were strongly correlated with the dDDH values (R^2^ = 0.9947). Based on the simulated exponential equation f(x) = 89.78*exp.(0.00107*x)-57.74*exp.(− 0.07575*x) for the entire dataset, the 70% dDDH threshold for species delineation corresponded to an ANI value of 96.48%, while ANI values of 95–96% corresponded to dDDH values of 59.193 to 66.29%, respectively (Additional file 5). This indicates that the traditional 70% dDDH threshold for BCC species demarcation is more stringent.

**Fig. 4:**
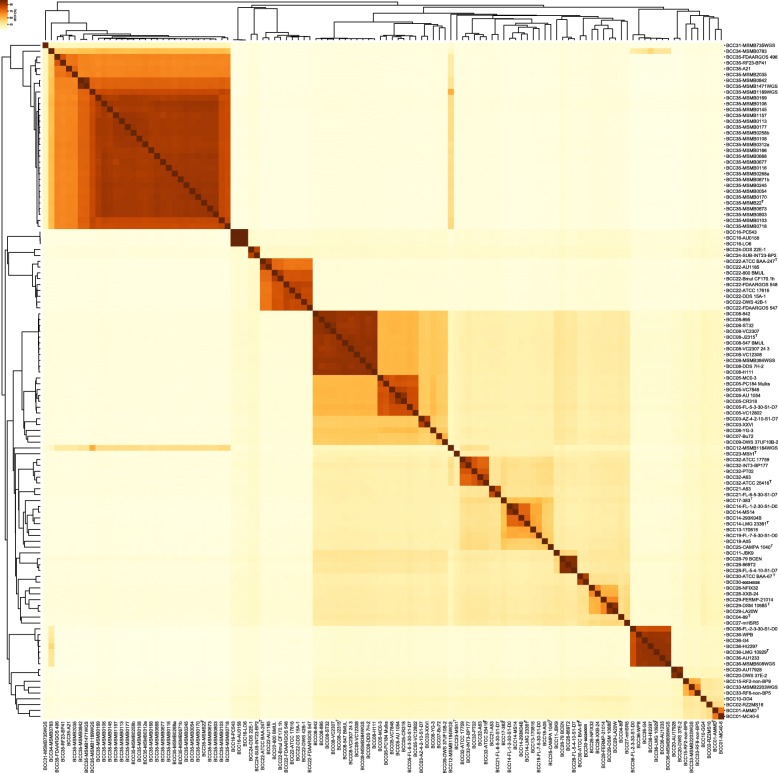
Heat map of pairwise dDDH values for 116 genomes of BCC strains. Hierarchical clustering of the 116 tested strains is indicated in both axes. At the 70% dDDH threshold for species delineation, 36 clusters or species (labeled BCC01-BCC36) are classified. Boxed regions indicate inferred clusters with at least two members. The name of each strain was composed of cluster labels and the original infraspecific name

Pairwise dDDH and ANI values were calculated and are shown in Fig. 3 and Additional file 4. Previous research has shown that the dDDH species cutoff (70%) is generally more stringent than the ANI species cutoff (95%~ 96%) [55]. Considering the intricacies of BCC taxonomy, we used the 70% dDDH (0.0361 for the recommended GGDC setting) and upper boundary 96% ANI (0.04 for the genome distance) thresholds to reclassify the BCC strains for species delineation, which divided 116 strains into 38 clusters and 36 clusters, respectively. All strains belong to the unanimous clusters except for the strain NFIX32 and FERMP-21014. Specifically, strain NFIX32 and XXB-24 shared a high mutual ANI value of 96.4% and dDDH value of 69.8%; although the mutual dDDH value is slightly below 70% threshold, the high ANI value (> 96% ANI threshold) and the well-constructed monophyly in species tree indicated they should be merged into one cluster that represents a novel species in BCC (Fig. 2). Similarly, strain FERMP-21014 shared dDDH value of 69.7% with *B. pyrrocinia* DSM 10685^T^ and were clustered into a different group according to the 70% dDDH threshold; however, they shared a high mutual ANI value of 96.4% (> 96% ANI threshold) and formed a highly supported clade in species tree (Fig. 2), which indicated that they should be merged into a single cluster. As a result, the 116 strains are reclassified into 36 clusters, labeled BCC01 through BCC36 (Fig. 4). Taking the type strain as the standard, we found that clusters BCC01, BCC04, BCC08, BCC14, BCC17, BCC22–23, BCC25, BCC29, BCC30, BCC32, BCC35 and BCC36 corresponded to the species *B. ambifaria*, *B. catarinensis*, *B. cenocepacia*, *B. contaminans*, *B. lata*, *B. multivorans*, *B. paludis*, *B. puraquae*, *B. pyrrocinia*, *B. stabillis*, *B. cepacia*, *B. ubonensis* and *B. vietnamiensis* well (Figs. 3 and 4). However, the taxonomy of the BCC strains was complicated and required further investigation.

### Reclassification of the BCC based on species tree and dDDH/ANI

**Fig. 5:**
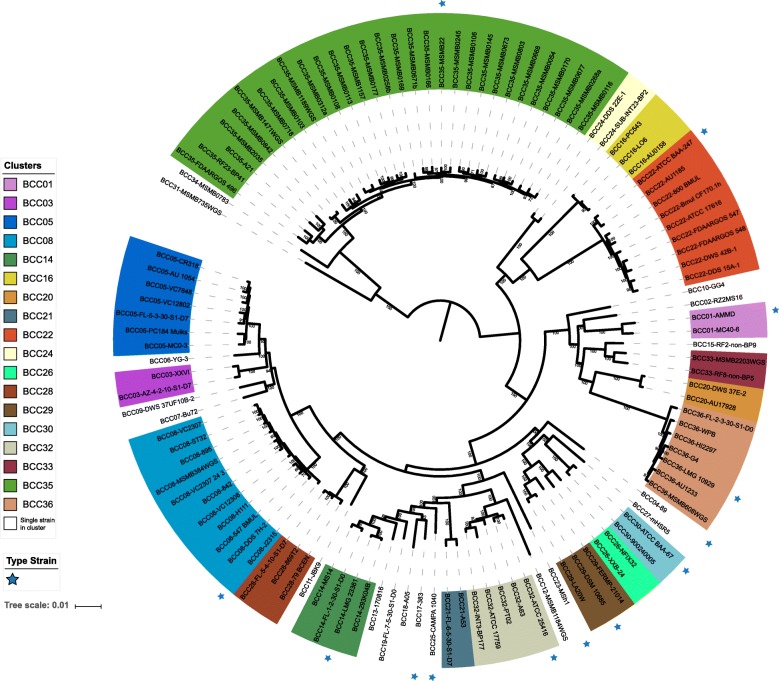
Reclassification of 116 BCC genomes.The maximum-likelihood phylogenetic tree is the same tree as in Fig. 2a except the layout is a circle instead of a rectangular phylogram. Leaves are colored according to their affiliation with clusters. (i.e., *Burkholderia cepacia* complex, BCC) and with at least two members; clusters with single members have a white background. Type strains are printed in bold font as well as marked by a blue star. Node support values from the Shimodaira-Hasegawa test are not shown if below 50%. The backgrounds are colored according to the corresponding cluster

To better classify the BCC species and elucidate their relationship with the BCC clusters, we annotated 36 clusters on our species tree, which were estimated based on single copy orthologous genes (Fig. 5). Through this approach, we redefined the classification and clarifies all misidentifications of the BCC.

The reclassified taxonomy of BCC species is well consistent with the species tree topology with high support, suggesting that our core-genome species tree agrees with the pangenome-based taxonomy (i.e., dDDH/ANI-based clustering) and is suitable for comprehensive taxonomic analysis in the BCC (Fig. 5).

We found that the previously identified *B. cepacia* strains excluding the misidentified strains LO6 and DDS 7H-2 (Fig. 2) were distributed in six clusters (BCC07, BCC09–12 and BCC32). Cluster BCC32 represented *B. cepacia* as indicated by the presence of type strain ATCC 25416^T^. Notably, strain A63 in the BCC32 strains that was misidentified as *B. territorii* before and should be reclassified to *B. cepacia*. Clusters BCC07 and BCC09–12 each had a single member located far away from the type strain cluster BCC32 (Fig. 5). These five strains diverged so much that they may represented five separate novel species in the BCC rather than *B. cepacia*. This suggested that the current taxonomy of *B. cepacia* is not well elucidated, which signified a need for further division of previously identified *B. cepacia* species.

As for *B. cenocepacia*, cluster BCC08 should be the representation due to the presence of the type strain *B. cenocepacia* J2315^T^ (=LMG 16656^T^). Again, we noted that strain DDS 7H-2 in cluster BCC08 was misidentified as *B. cepacia* and should be reclassified as *B. cenocepacia* on the basis of dDDH and ANI as well as phylogenetic analysis (Figs. 3 and 5). Specifically, BCC08 and BCC05 should represent *B. cenocepacia* genomovars IIIA and IIIB, respectively [23, 56, 57]. The dDDH and ANI estimations were above 79.8 and 97.7% among the cluster BCC05 and even above 89.2 and 98.8% within cluster BCC08, respectively. Between clusters BCC05 and BCC08, the dDDH values ranged from 59.7 to 60.9%, which is below the 70% threshold. In contrast, the ANI values ranged from 95.1 to 95.5%, which is near the threshold 95%~ 96% (Figs. 3 and 4). In the case of species delineation, dDDH is proven to be more discriminatory, as demonstrated in the study of *Vibrio cidicii* and *Bradyrhizobium brasilense* [58, 59]. Studies showed that when the species were compared against their closest relatives, ANI may be inconclusive, whereas the dDDH values were below the threshold [27]. Therefore, based on dDDH, clusters BCC05 and BCC08 should represent different but closely related species in the BCC. This finding indicated that the traditional *B. cenocepacia* genomovar IIIA represented classical *B. cenocepacia* and that genomovar IIIB should be divided as a novel species. Furthermore, BCC06, with only one strain formerly described as *B. cenocepacia*, should also be classified as a novel species because its dDDH and ANI estimations (56.5% and 94.4, respectively) with *B. cenocepacia* J2315^T^ were both lower than the threshold for species delineation (Figs. 3 and 5).

The clade containing strains previously identified as *B. stabillis* and *B. pyrrocinia* was confused. Cluster BCC30 contained two *B. stabillis* strains, including the type strain ATCC BAA-67^T^. Strains in cluster BCC26 yielded dDDH values ≤45.8% and ANI values ≤92.5% with type strain *B. pyrrocinia* DSM 10685^T^ and formed a separate branch in species tree (Figs. 3 and 5), indicating that they were previously misidentified and BCC26 should represent a putative novel species. Cluster BCC29 was represented by *B. pyrrocinia* DSM 10685^T^ and contained another strain (LA20W) that was previously misidentified as *B. stabillis*, which is also supported by another study [1]. Core genome phylogeny and dDDH/ANI similarity suggests that strain previously named as *B. stabillis* FERMP-21014 in BCC29 also should be reclassified as *B. pyrrocinia*, because it shared a middle dDDH value of 49.4% and ANI value of 93.3% with *B. stabillis* ATCC BAA-67^T^ that both lower than species delineation threshold. Cluster BCC27 (BCC27-mHSR5) in the clade represented a putative novel species that was previously misclassified as *B. pyrrocinia* as well. These results showed that traditional *B. pyrrocinia* species is more complicated than we thought and require further separation (Figs. 3, 4 and 5).

In cluster BCC35 representing *B. ubonensis*, strain FDAARGOS 496 was misclassified as *B. multivorans* (represented by BCC22). Cluster BCC34 contained only one strain that was formerly identified as *B. ubonensis*. However, this strain yielded a dDDH value of 59.2% and an ANI value of 95.7% with *B. ubonensis* MSMB22. Because dDDH is more discriminatory under such conditions, we believe that cluster BCC36 is likely to be a novel species that is closely related *B. ubonensis* (Fig. 3, 4 and 5).

In nine clusters (BCC03, BCC15–16, BCC20–21, BCC24, BCC28, BCC31 and BCC33), strains formed a monophyletic group, and their dDDH/ANI values satisfied the species delineation threshold. Despite a lack of a type strain, with the necessary reclassification of some isolates, these clusters probably represented *B. anthina*, *B. diffusa*, *B. dolosa*, *B. latens*, *B. metallica*, *B. pseudomultivorans*, *B. seminalis*, *B. stagnalis* and *B. territorii*, respectively.

Four clusters (BCC02, BCC13, BCC18 and BCC19), each formed by one strain, should be reclassified as four different putative new species.

Collectively, the current BCC species can be divided into 36 clusters. Twenty-two of the 36 clusters (BCC01, BCC03, BCC04, BCC08, BCC14–16, BCC17, BCC20–25, BCC28, BCC29, BCC30–33, BCC35 and BCC36) defined the current 22 known species with the appropriate correction of some strains. The other fourteen clusters (BCC02, BCC05–07, BCC09–13, BCC18–19, BCC26, BCC27 and BCC34) should be reclassified as 14 potential novel species (Fig. 5**,** Additional file 1).

## Discussion

In the past, taxonomic studies of *Burkholderia cepacia* complex have almost always been based on single markers or a small number of genes. In-depth taxonomic studies, especially for controversial groups such as BCC, should use the maximum resolution available: whole genome data. Whole genome sequences provide insight into the genetic nature of microbial species, yield new and superior tools for delineating bacterial species and for studying their phylogeny [60].

Phylogeny based on single-copy orthologous genes have been proven reliable in many studies involving bacteria, fungi, and plants [32, 61–63]. Our inferred species tree of BCC reconstructed by these markers provided us with an accurate phylogenetic relationship. Together with dDDH and ANI, two methods that are useful for species demarcation and are powerful in BCC species differentiation [1, 64, 65], we delineated BCC species as 36 clusters. The results showed that the 22 currently known species correspond to 22 of these clusters (Fig. 5). The other 14 clusters were reclassified as novel BCC species. New BCC species are continuing to be defined. Examples are shown by *B. contaminans, B. lata* (former taxon K), *B. stagnalis, B. territorii* (former group B and L), and the recently described *B. catarinensis* (formerly *Burkholderia sp*. 89) [1, 19, 20]. Hence, these clusters or putative novel species, at least somewhat, might be formally described and validly named in the near future.

Our study emphasized that there are great conflicts between traditional taxonomy and phylogeny in species classification, especially in species complexes such as BCC. Our dDDH/ANI clustering analysis suggested that the current taxonomy of BCC should be curated in a whole genomic view. For comparison, we also annotated the reclassified clusters based on *recA*, *hisA* and MLSA phylogeny (Additional files 6 and 7). For example, dDDH/ANI analysis showed that former *B. cenocepacia* genomovars IIIA and IIIB likely belonged to different species. In addition, several other strains identified as *B. cepacia* GG4, *B. diffusa* RF2-non-BP9, etc. should also be reclassified (Fig. 5, Additional file 1).

The accuracy of our study was limited to the strains with available and relatively high-quality whole genome sequences. For instance, the taxonomy of *B. cenocepacia* genomovars III C and III D are not discussed, as there are no complete genomes available. With the increasing number of BCC genomes available, the clustering status may vary but would be more complete, thus improving our knowledge with regard to the diversity of BCC.

Overall, our results strongly suggested that core phylogeny based on single-copy genes, as well as pangenome-based dDDH/ANI clustering, would provide a more preferable framework for demarcating species. Conjunctive use of two approaches both considered the information of vertical evolution during speciation and the overall genomic similarity between strains. To be sure, seeking out minimal phenotypic characteristics that could distinguish species, though difficult for closely related organisms (BCC or other species complexes), would still be biologically significant and necessary for a species description.

## Conclusion

In the present study, through comparison, we showed that 16S rRNA, *recA*, *hisA* and MLSA have limited power or resolutions in the taxonomic study of closely related bacteria like BCC. Using whole genome data, we divided current BCC species into 36 clusters and recognized all the misidentified or misclassified BCC isolates. With appropriate correction and reclassification, 22 of the 36 clusters defined current 22 known species. And the other 14 clusters should be reclassified as 14 potential novel species.

This is the first large-scale and systematic study of the taxonomic status of the BCC and could contribute to further insights into BCC taxonomy. Our analysis suggested the current taxonomy of BCC strains should be re-curated in a whole genomic view. And conjunctive use of core phylogeny based on single-copy orthologous genes, as well as pangenome-based dDDH/ANI clustering would provide a preferable framework for demarcating closely related species. As in this way, we both considered the information of vertical evolution during speciation and the overall genomic similarity between strains.

## Reviewer’s comments

Xianwen Ren PhD, School of Life Sciences, Peking University

**Reviewer summary:**

Differentiation of species within the *B. cepacia* complex is particularly problematic because of the highly similar phenotype. In this paper, Jin et al. conducted a large-scale and systematic study of the taxonomic status of the bacteria within the *B. cepacia* group using whole-genome sequences. Their results showed that the conjunctive use of core phylogeny based on single-copy orthologous genes, as well as pan-genome-based dDDH/ANI clustering would provide a preferable framework for demarcating closely related species. In addition, they reclassified several mis-classified BCC isolates and predicted 14 potential novel species. This work is interesting because of the debate over the appropriate way to delimitate species within bacterial species complex. This manuscript is the first taxonomic study using whole-genome sequences to discriminate bacterial species within the *B. cepacia* group, and is likely to provide further insights into their phylogeny and adaptation to diverse environments. The paper can be considered for publication with some minor revisions.

Author’s response: *We thank Dr. Xianwen Ren for his overall very positive review.*

**Reviewer recommendations to authors:**

**Minor concerns:**

1) All figures, especially Figure 1 and Figure 2, are too large. Please consider resize these illustrations while maintain the resolution.

Author’s response: *Thanks, we scaled* Figure 1*,* Figure 2 *and* Figure 5 *so as they could in accordance with A4 size.*

2) In Figure 3, values in the heatmap are illegible. A supplementary table is recommended.

Author’s response: *Good suggestion. we added pairwise dDDH and ANI values of 116 BCC strains in a table as additional file* Additional file 4*.*

3) ANI clustering results should be used to validate the species demarcation and compare the differences if exits.

Author’s response: *Thanks, based on the correlation between dDDH and ANI in additional file* Additional file 5*, 70% dDDH value correspond to approximate 96% ANI value. As our practice, the cluster results using the two threshold are almost the same except for two strains NFIX32 and FERMP-21014 (*Additional file 1*). NFIX32 and FERMP-21014 shared dDDH values 69.8 and 69.7% with their nearest neighbors XXB-24 and DSM 10685*^*T*^*respectively. Hence, they were divided into different groups according to the 70% dDDH threshold. However, strain NFIX32 and FERMP-21014 shared ANI value of 96.4% with XXB-24 and DSM 10685*^*T*^*respectively, which are greater than the upper bound 96% of ANI threshold. Considering their dDDH values are slightly below 70% threshold and the strains formed well monophyletic clades in species tree, we merged them to the clusters represented by their nearest neighbors. The detailed discussion can be found in the paragraph 2 of section****BCC species demarcation based on dDDH and ANI****in our revised manuscript.*

4) In the MLSA phylogeny, the authors chose three strains as outgroups while there is no outgroup strains were used in the species tree. The authors should add more explanations for such differences.

Author’s response: *Thanks, if outgroup strains were not included, more single copy orthologous genes shared by BCC can be inferred. As we focused on the relationships of strains inside BCC, more single copy orthologous genes within this group would help us to achieve maximum resolution. For parallel comparison, we root the species tree with the similar position of the MLSA phylogeny.*

5) L423 to L428. The combined representation of clusters such as “BCC26/BCC27” and “BCC30/BCC31” should be explained in detail.

Author’s response: *Many thanks to this suggestion. Based on the discussion from L338 to L365 in our manuscript, BCC26 and BCC27 as well as BCC30 and BCC31 should be merged. In our previous version of manuscript, we use “BCC26/BCC27” and “BCC30/BCC31” to represent the merged relationships. The term BCC26/BCC27 and BCC30/BCC31 in deed confuse readers. In our revised manuscript, we re-describe the species demarcation results as 36 clusters instead of 38 and removed the these combined representations.*

6) Gene names should be italic, such as recA and hisA in L21 and L25 of the abstract.

Author’s response: *Correction made as suggested.*

7) The organization of the paper can be improved and the Discussion section is too long.

Author’s response: *Thanks, we re-organized some results and included more detailed description in the section****BCC species demarcation based on dDDH and ANI****(L341 to L366) and section****Reclassification of the BCC based on species tree and dDDH/ANI****(L428 to L443). As reviewer’s suggestion, we deleted some redundant content and shorten the discussion section.*

## Supplementary information

**Additional file 1.** Complete list of the 116 strains used in this study with detailed information.

**Additional file 2.** List of the six strains that failed in the extraction of the full length of 16S rRNA gene sequences.

**Additional file 3.** Accession no. list of the additional eight 16S rRNA sequences.

**Additional file 4. **Pairwise dDDH values and ANI values list between 116 strains of the *B. cepacia* complex.

**Additional file 5.** Correlation analysis between dDDH values and ANI values. The exponential equation [y = 89.78*exp. (0.00107*x)-57.74*exp.(− 0.07575*x)] was obtained using a nonlinear simulation analysis method with the default option of the Curve Fitting Tool implemented in MATLAB R2018a. The two approaches revealed a significant correlation, with an r2 = 0.9947.

**Additional file 6.** Single-marker phylogenies of reclassified 116 BCC genomes. The trees are the same as in Fig. 1. The strains are labeled as cluster tags with the original infraspecific name.

**Additional file 7.** Species tree and MLSA of 116 reclassified 116 BCC genomes. The trees are the same as in Fig. 2. The strains are labeled as cluster tags with the original infraspecific name.

## Data Availability

The datasets supporting the conclusions of this article are available in the NCBI Genome Database, https://www.ncbi.nlm.nih.gov/genome. Accession numbers of sequences can be found in additional files.

## Abbreviations

ANI: Average nucleotide identityd
BCC: *Burkholderia cepacia* complex
CF: Cystic fibrosis
DDH: Digital DNA:DNA hybridization
DDH: DNA:DNA hybridization
GGDC: Genome-to-Genome Distance Calculator
GGDs: Genome-to-Genome Distances
MLSA: Multilocus sequence analysis
MLST: Multilocus sequence typing
WGS: Whole genome sequencing
