# Supplementary figures and images for: Genome-based classification of Burkholderia cepacia complex provides new insight into its taxonomic status
Source: Biol Direct. 2020 Mar 4;15:6. doi: 10.1186/s13062-020-0258-5 (PMC7057466; doi:10.1186/s13062-020-0258-5)

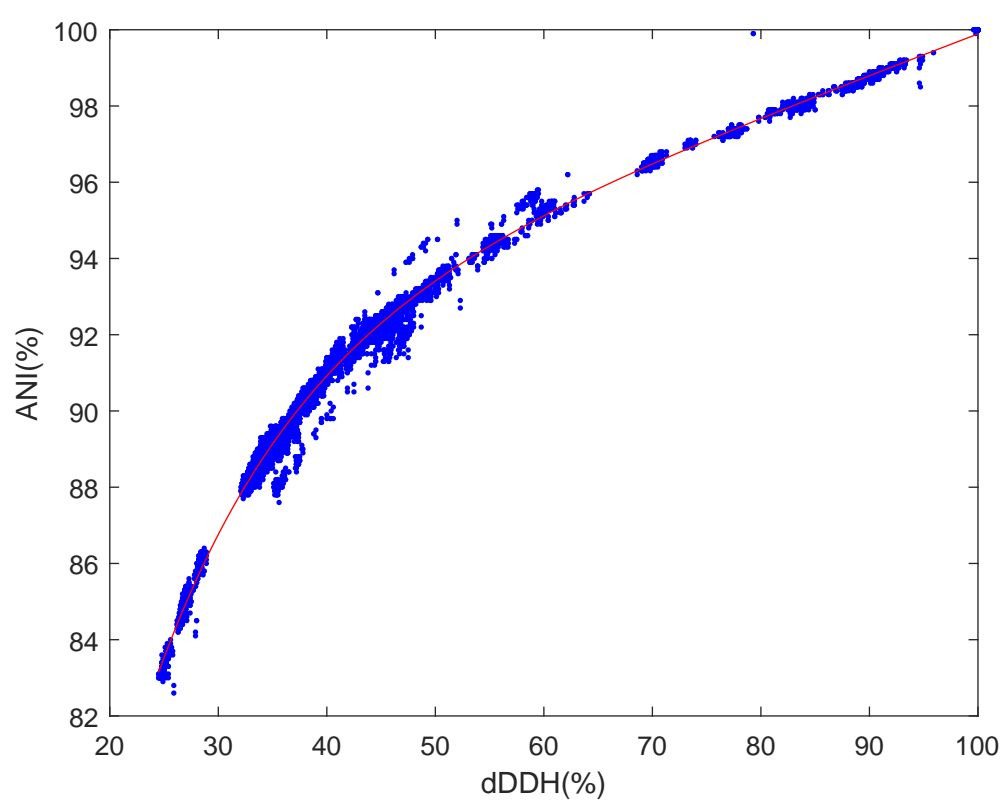

Supplement: Supplementary file 5 — Additional file 5. Correlation analysis between dDDH values and ANI values. The exponential equation [y = 89.78*exp. (0.00107*x)-57.74*exp.(− 0.07575*x)] was obtained using a nonlinear simulation analysis method with the default option of the Curve Fitting Tool implemented in MATLAB R2018a. The two approaches revealed a significant correlation, with an r2 = 0.9947. [file 13062_2020_258_MOESM5_ESM.pdf]

A

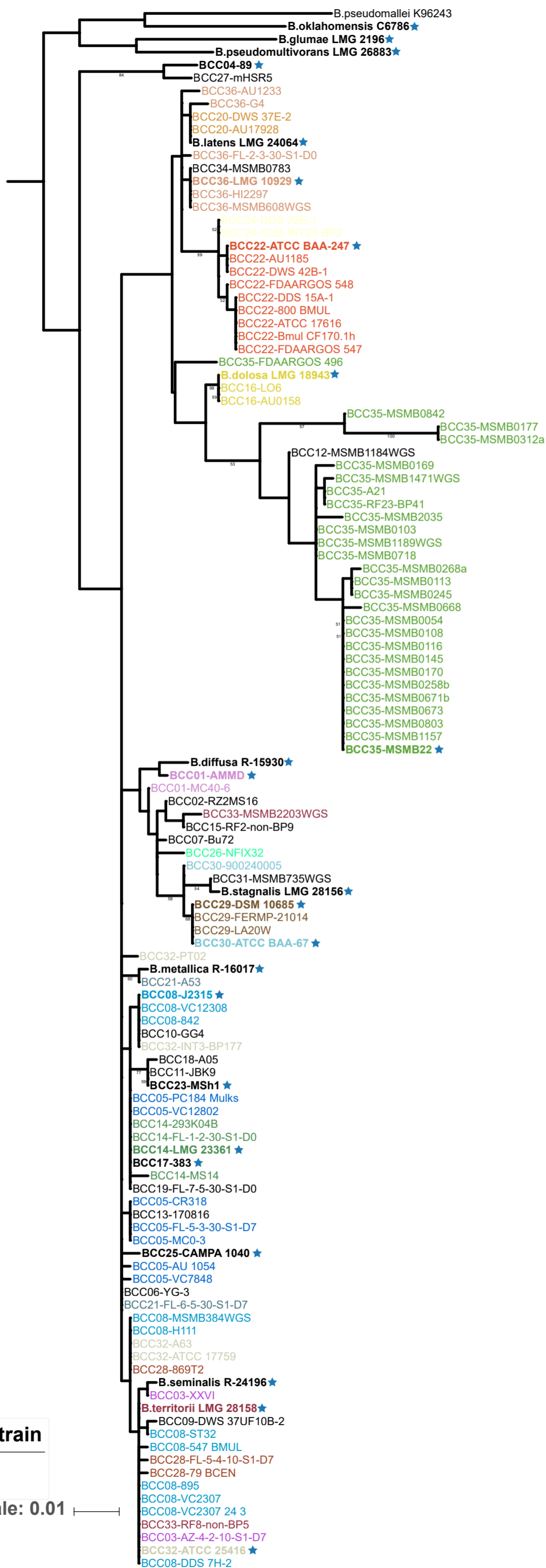

B

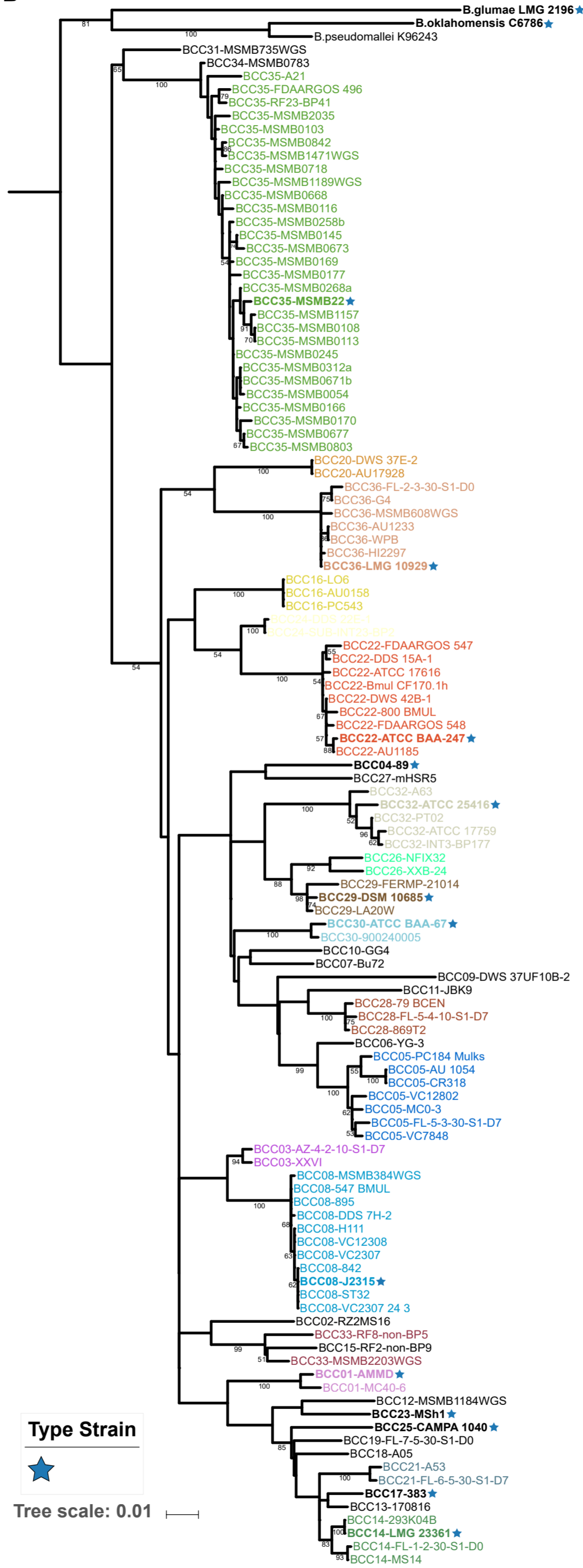

C

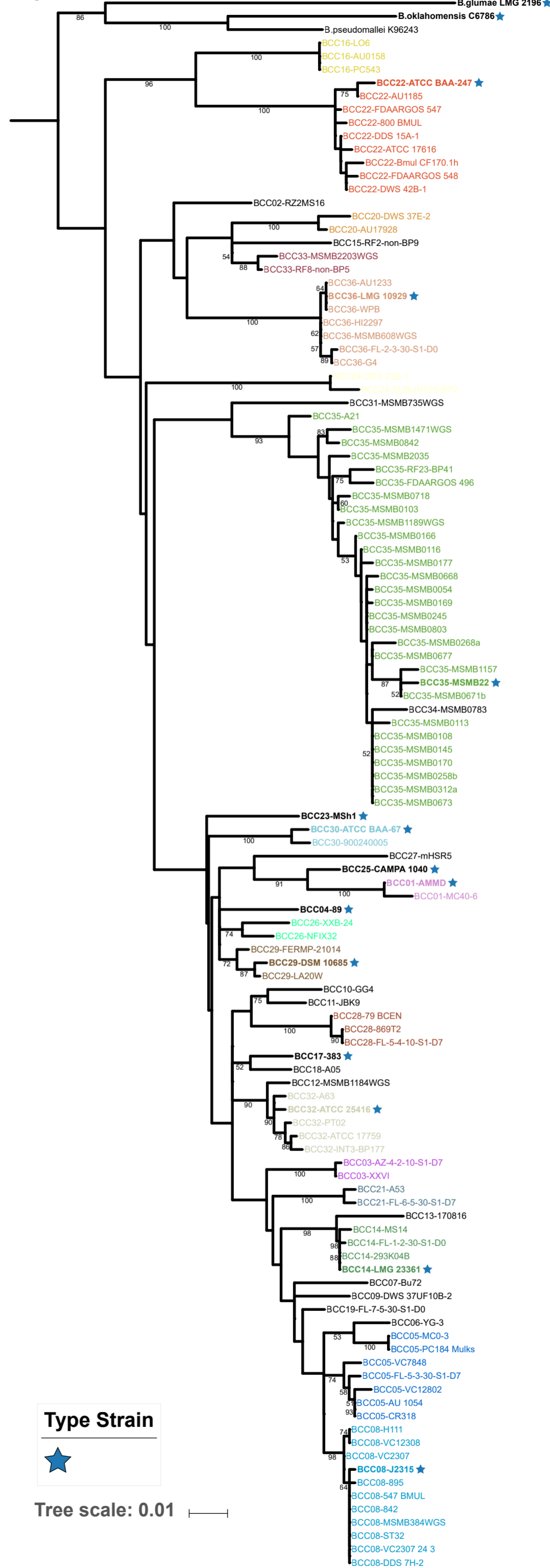

Supplement: Supplementary file 6 — Additional file 6. Single-marker phylogenies of reclassified 116 BCC genomes. The trees are the same as in Fig. 1. The strains are labeled as cluster tags with the original infraspecific name. [file 13062_2020_258_MOESM6_ESM.pdf]

A

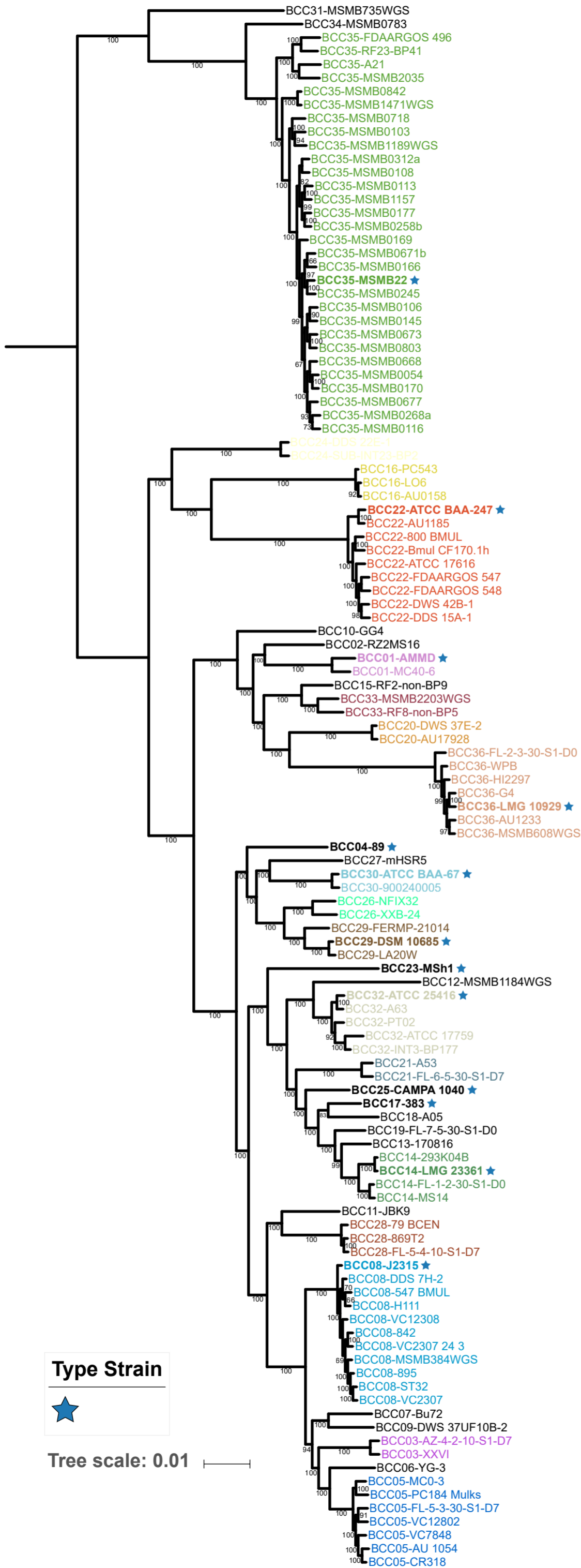

B

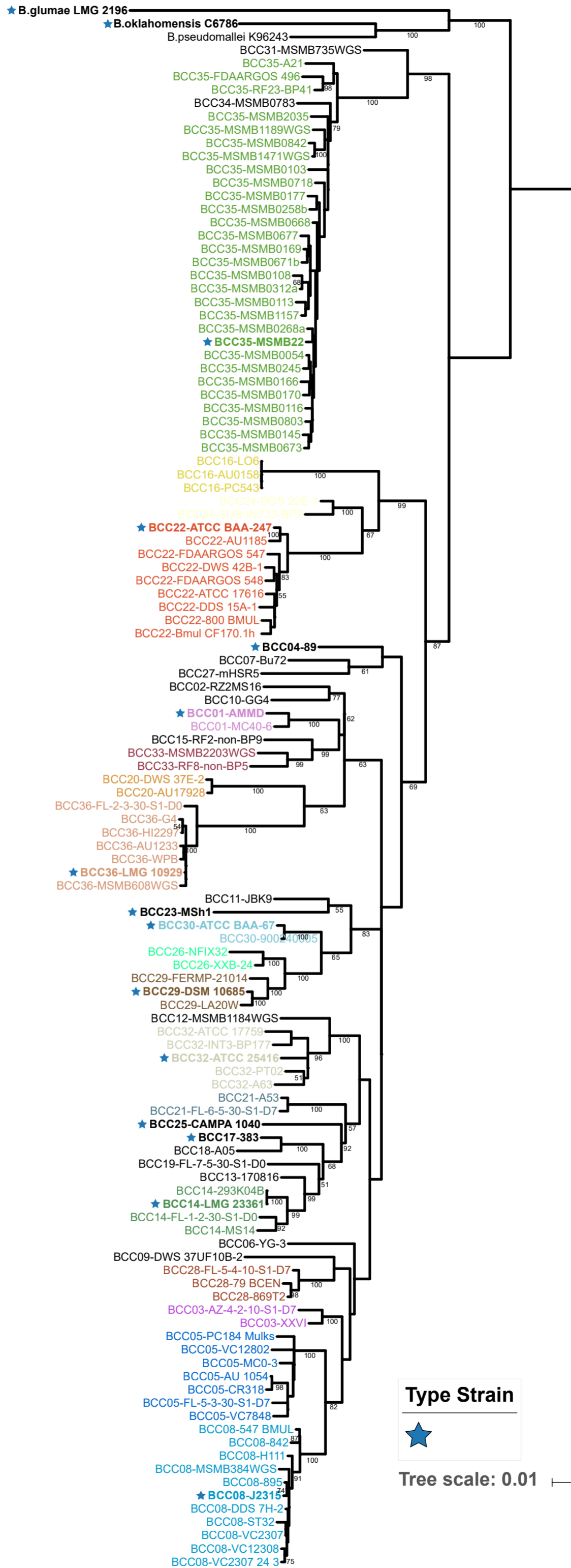

Supplement: Supplementary file 7 — Additional file 7. Species tree and MLSA of 116 reclassified 116 BCC genomes. The trees are the same as in Fig. 2. The strains are labeled as cluster tags with the original infraspecific name. [file 13062_2020_258_MOESM7_ESM.pdf]
